# Systems approach to the study of stretch and arrhythmias in right ventricular failure induced in rats by monocrotaline

**DOI:** 10.1016/j.pbiomolbio.2014.06.008

**Published:** 2014-08

**Authors:** David Benoist, Rachel Stones, Alan P. Benson, Ewan D. Fowler, Mark J. Drinkhill, Matthew E.L. Hardy, David A. Saint, Olivier Cazorla, Olivier Bernus, Ed White

**Affiliations:** a Multidisciplinary Cardiovascular Research Centre, University of Leeds, UK; b L′Institut de Rythmologie et Modelisation Cardiaque, INSERM U1045, Université de Bordeaux, France; c Faculty of Life Sciences, University of Manchester, UK; d School of Medical Sciences, University of Adelaide, Australia; e INSERM U1046, Université Montpellier 1, Université Montpellier 2, France

**Keywords:** Systems Biology, Pulmonary artery hypertension, Mechanosensitivity, Arrhythmias, Monocrotaline

## Abstract

We demonstrate the synergistic benefits of using multiple technologies to investigate complex multi-scale biological responses. The combination of reductionist and integrative methodologies can reveal novel insights into mechanisms of action by tracking changes of *in vivo* phenomena to alterations in protein activity (or *vice versa*). We have applied this approach to electrical and mechanical remodelling in right ventricular failure caused by monocrotaline-induced pulmonary artery hypertension in rats.

We show arrhythmogenic T-wave alternans in the ECG of conscious heart failure animals. Optical mapping of isolated hearts revealed discordant action potential duration (APD) alternans. Potential causes of the arrhythmic substrate; structural remodelling and/or steep APD restitution and dispersion were observed, with specific remodelling of the Right Ventricular Outflow Tract. At the myocyte level, [Ca^2+^]i transient alternans were observed together with decreased activity, gene and protein expression of the sarcoplasmic reticulum Ca^2+^-ATPase (SERCA). Computer simulations of the electrical and structural remodelling suggest both contribute to a less stable substrate.

Echocardiography was used to estimate increased wall stress in failure, *in vivo*. Stretch of intact and skinned single myocytes revealed no effect on the Frank-Starling mechanism in failing myocytes. In isolated hearts acute stretch-induced arrhythmias occurred in all preparations. Significant shortening of the early APD was seen in control but not failing hearts. These observations may be linked to changes in the gene expression of candidate mechanosensitive ion channels (MSCs) TREK-1 and TRPC1/6. Computer simulations incorporating MSCs and changes in ion channels with failure, based on altered gene expression, largely reproduced experimental observations.

## 1 Introduction

### 1.1 Reduction and integration

While Systems Biology has been interpreted by some as confined to the study of genes and proteins, there is no necessity for such a narrow definition (Kohl et al., 2010, Kuster et al., 2011). In addition the overall goals of Systems Biology seem similar to those long held by Integrative Physiologists (Greenhaff and Hargreaves, 2011). The approach described here is in keeping with ideas presented in the above articles. It stems from the self-evident conviction that combining technologies to allow a reductionist (organism to gene) and/or integrative approach (gene to organism) to the study of a complex problem, such as heart failure, will reveal more information about ‘the system’ than study at a single level.

### 1.2 The right ventricle and pulmonary artery hypertension

Pulmonary artery hypertension (PAH) is a progressive disease caused by vasoconstriction and/or cell proliferation in the pulmonary vasculature. Despite new agents that specifically target the vasculature (Rhodes et al., 2009), median survival time is 5–6 years (Naeije, 2010, Galie et al., 2009). Serious consequences of increased pulmonary artery pressure are right heart hypertrophy, dilatation, arrhythmias and failure. Right ventricular (RV) failure is the major cause of death in sufferers of PAH (Haddad et al., 2008, Bogaard et al., 2009), however the RV is not currently a target for therapy, other than by the supportive use of diuretics and digoxin (Galie et al., 2009). There is an acknowledged need for further study of the failing RV (Haddad et al., 2008, Bogaard et al., 2009, Voelkel et al., 2006).

Electrical remodelling is seen in patients with PAH, for example increased QT interval and time from T-wave peak to end (Hlaing et al., 2005) and the association between QT prolongation and increased mortality (Rich et al., 2013). The RV is anatomically and functionally distinct from the left ventricle (LV) and works under different mechanical conditions. The thin wall of the RV makes it susceptible to distension by sudden increases in venous return (Bristow et al., 1998, Mebazaa et al., 2004, Voelkel et al., 2006). Mechanical stimuli, such as increased stress, due to elevated afterload and strain due to diastolic chamber dilation are predicted to be important factors in the progression to RV failure in PAH (Bristow et al., 1998, Mebazaa et al., 2004). In support of this idea, chronic pharmacological preload reduction in mice was shown to prevent the development of exercise-induced arrhythmogenic right ventricular cardiomyopathy (Fabritz et al., 2011).

### 1.3 MCT model

The monocrotaline (MCT) model of PAH in rats is well established. MCT is a pyrrolizidine alkaloid from the plant *Crotalaria spectabilis.* A single injection of MCT (60–80 mg/kg) causes injury to the vascular endothelium of the lung, hypertrophy of the pulmonary vasculature and pulmonary hypertension (Kay et al., 1967). Compensated RV hypertrophy occurs after 2–3 weeks and right heart failure after 3–5 weeks (Hardziyenka et al., 2006, Campian et al., 2006, Bogaard et al., 2010, de Man et al., 2012). We have reported the model has a pro-arrhythmic substrate (Benoist et al., 2011, Benoist et al., 2012) and it shares some electrical remodelling characteristics with human patients (see (Benoist et al., 2011, Benoist et al., 2012, Hlaing et al., 2005, Rich et al., 2013). There is evidence for a chronic increase in wall stress (Stones et al., 2013).

### 1.4 Alternans and arrhythmias

Alternans are known to be precursors of serious arrhythmias (Cutler and Rosenbaum, 2009). They manifest as beat to beat variations in amplitude of the T-wave in the ECG (Qu et al., 2010), in action potential duration (APD) and/or [Ca^2+^]i transient amplitude (Laurita and Rosenbaum, 2008, Cutler and Rosenbaum, 2009, Weiss et al., 2011). They may be the consequence of steep APD restitution and/or dysfunction in Ca^2+^ handling (Pruvot et al., 2004). In tissue, alternans can be concordant, when adjacent regions alternate in phase or be discordant where regions are out of phase. The dispersion of repolarisation created by discordant alternans makes them more likely to descend into serious arrhythmias.

### 1.5 Mechanical stimulation and arrhythmias in the heart

Mechanical stimuli, such as stress and strain, have important effects on the normal and diseased heart. Acute increases in cardiac chamber volume stretch the myocardium and increase its contractility (the Frank-Starling law of the heart) and the intrinsic beating frequency of the cardiac pacemaker (the Bainbridge effect). Chronic increases in chamber volume or wall stress, caused e.g. by hypertension, trigger compensatory hypertrophy but ultimately lead to heart failure. Acute stretch can also trigger arrhythmias in both ventricles (Franz et al., 1992) and atria (Bode et al., 2001) possibly via the activation of mechanosensitive ion channels (MSCs) see articles in (Kohl and Ravens, 2003, Kohl et al., 2005) and (White, 2006).

There is some evidence to suggest that stretch-activated arrhythmias are more prominent in diseased hearts, e.g. in atrial fibrillation (Bode et al., 2001), heart failure (Wang et al., 1994) and compensated hypertensive hypertrophy (Salmon et al., 1997). This may be due to changes in MSC activity and/or a pro-arrhythmic state in disease, caused by changes such as altered dispersion of repolarisation (Antzelevitch, 2005). However, given the important role stress and strain play in many cardiac dysfunctions, surprisingly few studies have investigated their acute roles in the modulation of mechanical and electrical activity in diseased tissue.

### 1.6 Mechanosensitive ion channels

MSCs are activated by increased tension in the lipid membrane and/or the cytoskeleton (Hamill and McBride, 1996, Hamill and Martinac, 2001). In the heart there is evidence for 2 major types of MSC (excluding volume-regulated channels); K^+^-selective MSCs (MSC_K_) and non-specific cationic MSCs (MSC_NS_) which conduct Na^+^ and Ca^2+^ (Kohl and Ravens, 2003, Kohl et al., 2005, White, 2006). MSC_K_ appears to include TREK-1 (Xian et al., 2006, Kelly et al., 2006) a member of the 2 pore–domain K^+^ channel family (Patel and Honore, 2005). It has been reported that the levels of TREK-1 protein in the heart can be increased in systemic hypertension (Cheng et al., 2006) and by acute stretch (Zhao et al., 2007).

Although MSC_NS_ is highly implicated in the effects of acute stretch (Kohl and Ravens, 2003, Kohl et al., 2005, White, 2006) their identity is uncertain. Research has centred upon Transient Receptor Potential (TRP) channels, since (Maroto et al., 2005) identified TRPC-1 as a vertebrate MSC_NS_ (Barritt and Rychkov, 2005). Although this interpretation has been questioned with respect to channels expressed in cell lines (Gottlieb et al., 2008), the initial observation has sparked research in native tissue which has provided evidence for TRPC function in the heart (Ju et al., 2007) and as MSCs, in both skeletal (Yeung et al., 2005) and cardiac muscle (TRPC-6) (Dyachenko et al., 2009). TRPC-1 is increased in cardiac hypertrophy e.g. (Ohba et al., 2007) and TRPC-3 by stretch (Dalrymple et al., 2007). The potential involvement of multiple TRPC channels may be linked to channel heteromeric sub-unit structure and common function within a given cell (Beech, 2005, Dietrich et al., 2006).

MSCs are thought to modulate action potential shape and generate stretch-activated arrhythmias both by the generation of ion currents and in the case of MSC_NS_, the modulation of intracellular Ca^2+^, but there has been relatively few studies of MSCs in diseased tissue (Kohl and Ravens, 2003, Kohl et al., 2005). The spontaneously hypertensive rat is more susceptible to stretch-activated arrhythmias (Evans et al., 1995, Kim et al., 2012) via mechanisms associated with MSC_NS_ (Salmon et al., 1997) and these animals are reported to have a greater density of such currents (Kamkin et al., 2000).

We are interested in the electrical and mechanical remodelling that occurs in heart failure. This article will describe our investigation of two aspects of remodelling in the MCT model; the occurrence of alternans, a pre-cursor of serious arrhythmias and the acute effect of mechanical stimulation. Our aim is to demonstrate the utility and feasibility of a systems approach to understand mechanisms associated with a complex problem such as heart failure induced by PAH.

## 2 Methods

### 2.1 Monocrotaline model of PAH

Wistar rats (200 g) were either given a single injection of saline (CON) or a single injection of 60 mg/kg MCT to induce PAH and RV failure within 3–4 weeks (FAIL). Some animals were given a lower dose of 30 mg/kg MCT to induce a non-failing hypertropic phenotype (HYP) (Benoist et al., 2011, Benoist et al., 2012, Stones et al., 2013). When FAIL animals showed signs of heart failure (e.g. weight loss, lethargy) they were humanely killed, HYP and CON animals were killed on equivalent days. All experiments were performed with local ethical and UK Home Office approval.

Methods used in this study have been previously described, unless otherwise stated; optical mapping of electrical activity, myocyte Ca^2+^ transients and contraction, protein measurement by Western blotting and mRNA by real-time PCR in (Benoist et al., 2011, Benoist et al., 2012, Stones et al., 2013, Stones et al., 2009); force-pCa in permeabilised single myocytes (Cazorla et al., 2005); force-sarcomere length (SL) relationships in intact single myocytes (Calaghan and White, 2004). All experiments were performed at 37 °C unless otherwise stated.

### 2.2 Echocardiography

Echocardiography was performed on rats anaesthetised with 5% isofluorane (mixed with O_2_) and maintained at 1.5% during examination. Images were acquired with a GE Healthcare Vivid7 with a 10S probe at a frequency of 11.5 MHz. A non-invasive, *in vivo* estimate of RV wall stress was made: (*σ*) = Pr/2h where *P* = trans-wall pressure difference; *r* = radius of curvature and *h* = wall thickness. RV systolic pressure was estimated from pulmonary artery acceleration time (PAAT) using pulse wave Doppler (Jones et al., 2002). Radius of curvature of the RV was measured in the short axis in B mode and RV wall thickness in M mode.

### 2.3 Whole heart stretch and arrhythmia

Isolated hearts were Langendorff perfused at 37 °C with a bicarbonate-based physiological saline solution. The RV monophasic action potential duration (MAPD) was measured in hearts stimulated at 5 Hz, before and after an increase in RV volume by inflation of an indwelling fluid filled balloon. The balloon was connected to a pressure transducer allowing RV force to be measured. Stretch was achieved by inflating each RV to the individual volume that gave maximum active force development.

### 2.4 Ventricular structure

Ventricular structural remodelling was quantified using diffusion tensor magnetic resonance imaging (DT-MRI) as previously described (Benson et al., 2011). Briefly, fixed hearts were immersed in the perfluoropolyether Fomblin, then high-resolution (200 μm isotropic) imaging of fibre structure was performed using an NMR spectrometer with a 9.4 T magnet. Diffusion of protons was measured throughout the tissue in a set of 12 optimized directions using a three-dimensional diffusion-weighted spin-echo sequence with reduced encoding at 20 °C: repetition time: 500 ms; echo time: 15 ms; diffusion gradients with 2 ms duration and 7 ms separation; *b* = 1000 s/mm^2^. Diffusion tensors, and the eigenvectors of these tensors, were calculated from the diffusion measurements, before fibre structure was quantified, principally as the fibre inclination or helix angle, from these vectors using in-house software.

### 2.5 Computer simulations

For structural and electrical remodelling interactions, levels of expression of mRNA for individual ion channels and action potential profiles obtained from LV and RV of CON and FAIL rats (Benoist et al., 2011) were used to scale ion channel conductances in a single rat ventricular myocyte model (Pandit et al., 2001). These were then incorporated into heterogeneous and anisotropic 3D ventricle models (3 CON and 3 FAIL) with anatomy reconstructed from DT-MRI at 200 μm resolution (Benson et al., 2011).

To simulate our *in vitro* stretch experiments the adapted (Pandit et al., 2001) model was further modified to reproduce our *in vitro*-measured MAPDs at 25%, 50% and 90% repolarisation with no stretch (Fig. 9), by reducing *I*_to_, *I*_ss_ and *I*_Ks_ maximal conductances by 70% and increasing their activation time constants five-fold.

**Fig. 1:**
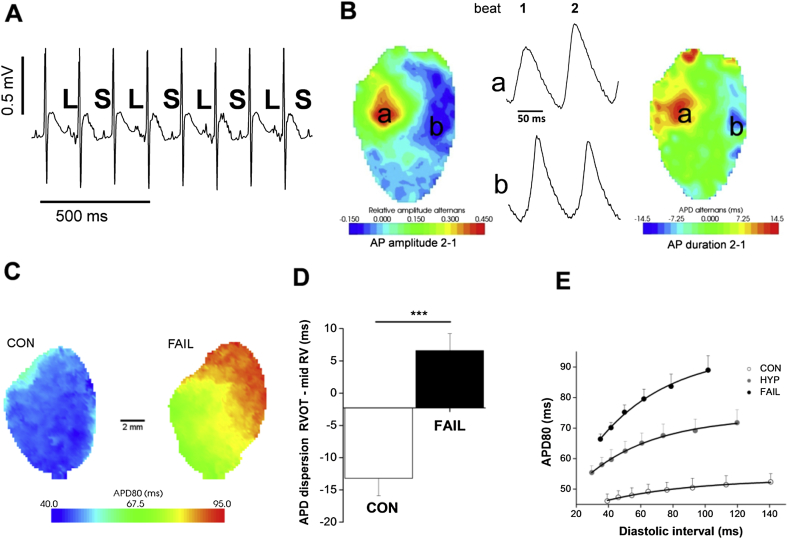
A. T-wave alternans from the ECG of an unrestrained, conscious, FAIL animal. The T-wave shows a long (L), short (S) pattern. B Discordant alternans in an isolated heart from a FAIL animal revealed by optical mapping. Differences between beat 1 and 2 in zones a and b are out of phase for both AP amplitude and AP duration. C. Increased APD and dispersion of APD in the RV of a FAIL animal compared to a CON animal. D Difference in APD_80_ between RVOT and mid-RV at 5 Hz. In CON hearts APD in the RVOT region was shorter than the mid-RV but longer in MCT treated hearts (***P < 0.001 *N* = 11 CON and 9 MCT hearts), thus APD remodelling was greater in the RVOT than the mid-RV. E APD restitution was significantly steeper in FAIL than CON hearts or hearts from animals with stable hypertrophy (HYP). A, B, C and E modified from (Benoist et al., 2012).

**Fig. 2:**
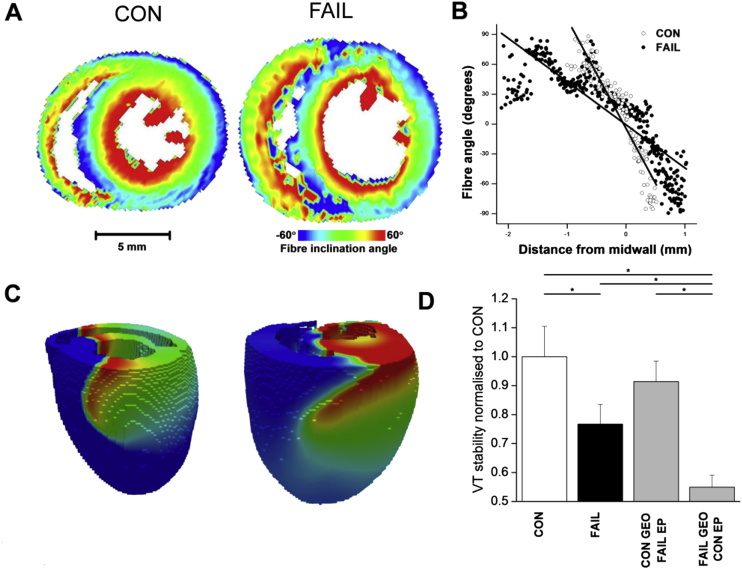
A Fibre angle assessed by DT-MRI in a CON and FAIL heart. B RV fibre angle plotted against distance from mid-wall, the change in fibre angle is slower and more varied in the FAIL heart which may indicate increased structural heterogeneity. C Snapshots showing simulated ventricular tachycardia (VT) in CON and FAIL models. Red is excited tissue, blue resting: waves are rotating anticlockwise. D VT was initiated in various ventricular locations (192 simulations in total) and stability (time to breakup, e.g. into fibrillation) measured. Stability of VT decreased in FAIL compared to CON. Restoring CON geometry (GEO) but maintaining FAIL electrophysiology (EP) recovered stability, whereas CON EP with FAIL GEO decreased stability further. *P < 0.05.

**Fig. 3:**
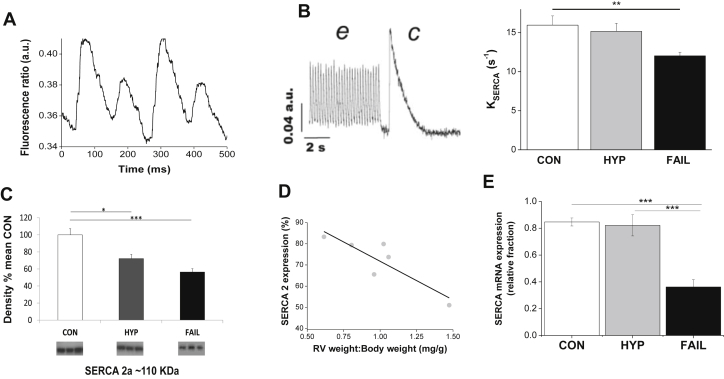
A Intracellular Ca^2+^-transient (Fura-4F fluorescence) alternans for a RV myocyte isolated from a FAIL animal stimulated at 9 Hz. B SERCA activity estimated as *K*_SERCA_ was significantly reduced in RV myocytes from FAIL animals. *K*_SERCA_ was calculated as the difference between the rate constant of decay of the electrically stimulated Ca^2+^-transient at 5 Hz (e, representing Ca^2+^ removal by SERCA and Na–Ca exchange) and caffeine stimulated Ca^2+^ transient (c, representing Ca^2+^ removal by Na–Ca exchange). C Levels of SERCA protein, estimated by Western blot, were significantly lower in HYP and FAIL RV myocardium than CON (data expressed as % of the mean density of CON samples, *N* = 6 in each group) D. In the HYP group SERCA density was significantly correlated with the RV weight:body weight ratio *R*^2^ = 0.7, *P* < 0.05). E Levels of mRNA for SERCA, estimated by real-time RT-PCR, were significantly lower in FAIL myocardium than HYP or CON (data expressed relative to a calibrator sample normalised to the housekeeper gene 18S, *N* = CON 12, HYP 7, FAIL 14). A and B modified from (Benoist et al., 2012). *P < 0.05; **P < 0.01; ***P < 0.001.

**Fig. 4:**
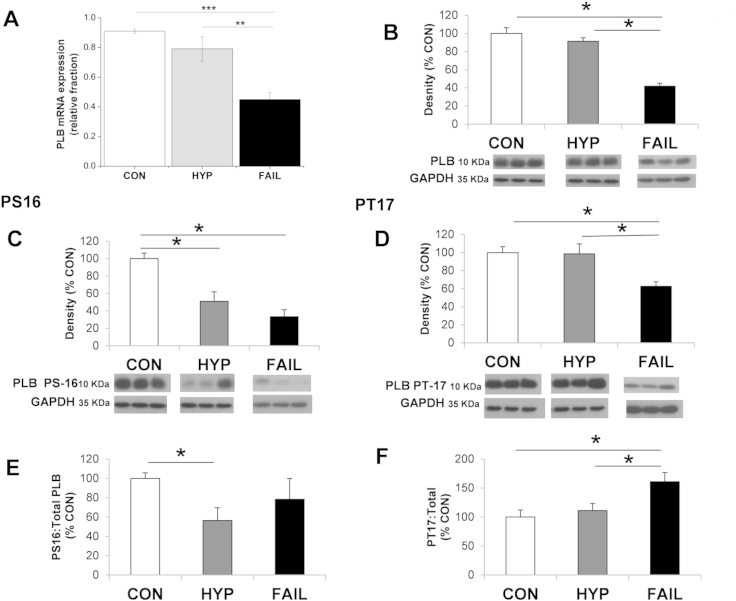
A There was a significant decrease in the level of mRNA for PLB In FAIL compared to HYP and CON myocardium, estimated by real-time RT-PCR, (data expressed relative to a calibrator sample and normalised to the housekeeper gene 18S (*N* = 12 CON, 7 HYP, 14 FAIL hearts). B There was a significant decrease in the level of total PLB protein in FAIL compared to CON and HYP myocardium hearts estimated by Western blot, PLB density normalised to GAPDH (*N* = 6 in each group). C Levels of protein for phosphorylated Serine 16 (c’AMP_dependent site) and D for phosphorylated Threonine 17 (Ca^2+^-calmodulin dependent site) were significantly reduced in FAIL compared to CON. E levels of phosphorylated Serine 16 and F of phosphorylated Theonine 17 were expressed as a % of total PLB. There was a statistically significant increase in phosphorylated Threonine 17: Total PLB for FAIL compared to CON in contrast the level of phosphorylated Serine 16: Total PLB did not change. *P < 0.05; **P < 0.01; ***P < 0.001.

**Fig. 5:**
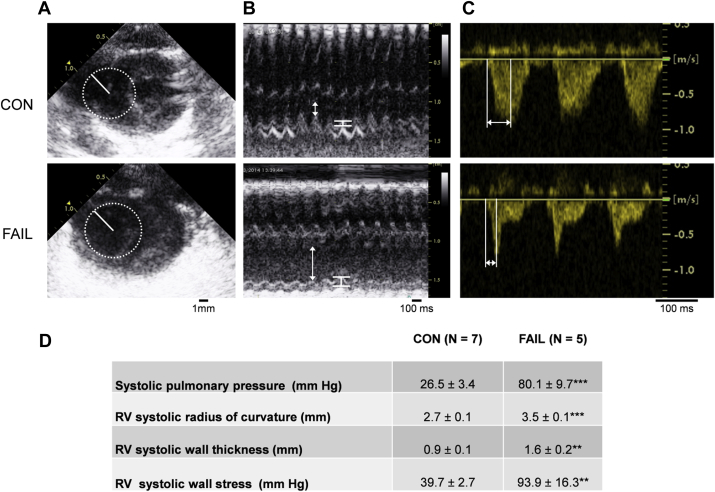
Systolic RV wall stress calculated using echocardiography. A Radius of curvature (solid line) was found by fitting a circle at the widest part of the junction between the RV free wall and septum during systole measured in B-mode. B RV wall thickness (white bars) and internal diameter (arrows) measured during systole in M-mode: hypertrophy and dilatation were evident in FAIL rats. C Pulmonary artery acceleration time (PAAT) measured as the time from onset to peak flow rate using pulsed-wave Doppler. PAAT was reduced in FAIL rats. D Mean values for RV systolic pressure, RV radius of curvature and RV wall thickness and calculated wall stress (see Methods) in 7 CON and 5 FAIL hearts. There was a significant increase in all parameters in FAIL compared to CON animals. **P < 0.01; ***P < 0.001.

**Fig. 6:**
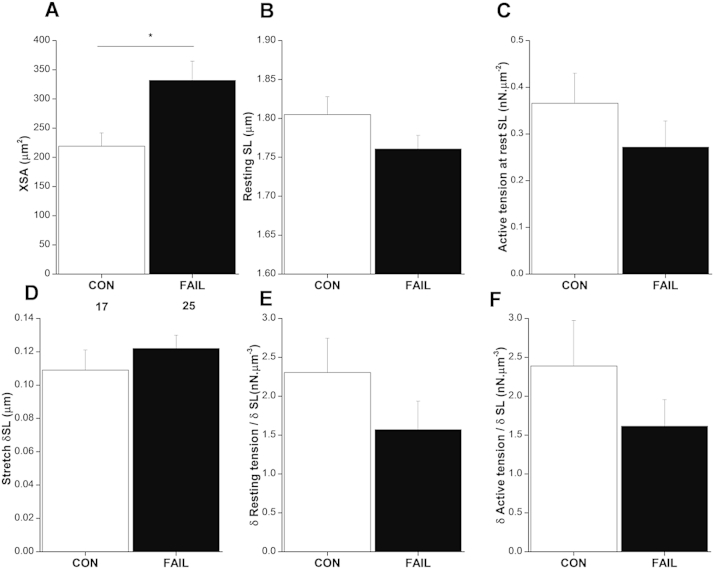
A Cross-sectional area (XSA) of RV myocytes from FAIL hearts was significantly larger than CON myocytes. There were no significant differences between CON and FAIL myocytes in: B resting sarcomere length (SL). C Absolute active force, normalised to myocyte XSA. D increase in SL in response to stretch E. increase in resting tension per unit stretch. F increase in active tension per unit stretch (*n* = 17 CON and 25 FAIL myocytes). *P < 0.05.

**Fig. 7:**
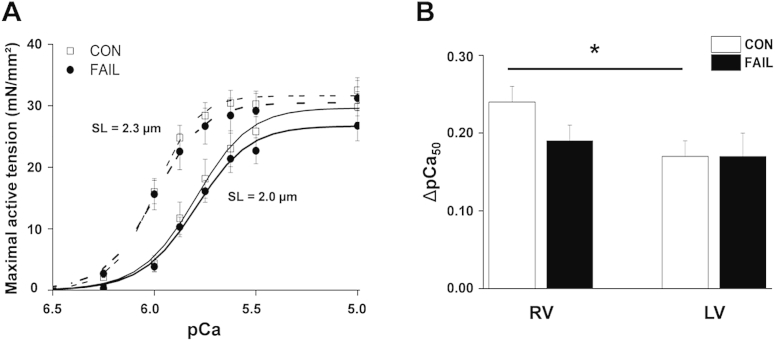
A Maximal active tension in response to alterations in pCa for triton skinned RV myocytes from CON and FAIL hearts. Experiments were performed at 25 °C. Data was collected at SL 2.0 and 2.3 μm and fitted to a Hill equation. Increased SL caused a left shift in the curves. B Change in the pCa50 (index of length-dependent increase in myofilament Ca^2+^- sensitivity) in response to an increase in SL from 2.0 to 2.3 μm for RV and LV myocytes from CON and FAIL hearts. There was a significant difference in ΔpCa50 between RV and LV in CON myocytes but no differences between CON and FAIL (CON RV *n* = 12; CON LV *n* = 12; FAIL RV *n* = 18; FAIL LV *n* = 14 from *N* = CON 3, FAIL 4 hearts). *P < 0.05.

**Fig. 8:**
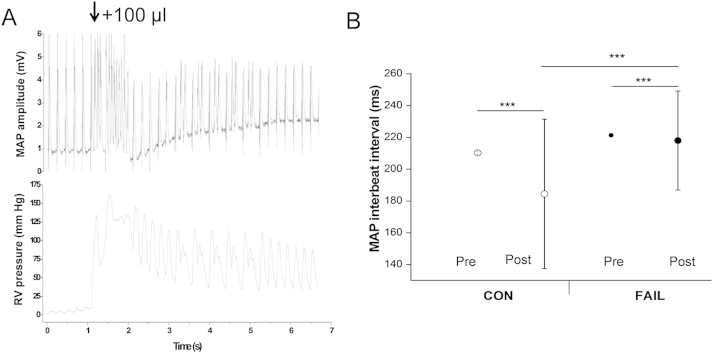
A Experimental traces showing the effect of inflating a balloon to 100 μl in the RV of a Langendorff perfused heart on the electrical (upper trace) and mechanical (lower trace) activity of the heart paced at 5 Hz. Inflation caused a brief disruption to the rhythm of the heart. B Interbeat interval before and immediately after balloon inflation, data show mean ± SD. There was a significant increase in the SD of the beat to beat interval in both CON and FAIL hearts, indicating a stretch-induced decrease in rhythmicity. The increase in SD was significantly greater in CON than FAIL hearts. (*N* = 6 CON, 8 FAIL hearts). ***P < 0.001.

**Fig. 9:**
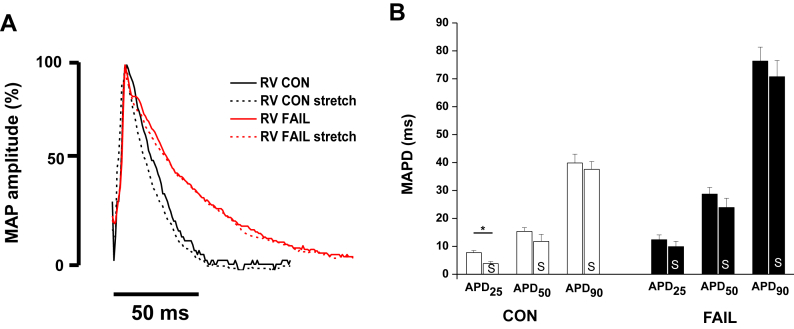
A representative monophasic action potential (MAP) traces from a CON and FAIL heart, % amplitude is shown to facilitate comparison of repolarisation. B MAP duration (MAPD) at 25, 50 and 90% repolarisation before (unmarked) and after (S) an increase in RV volume to that giving the maximal active tension in CON and FAIL hearts. Stretch caused a significant decrease in MAPD_25_ in CON hearts (*N* = 6 CON, 8 FAIL hearts). *P < 0.05.

MSC currents were incorporated as in (Healy and McCulloch, 2005). Currents were carried through TRPC1/6 (*IMSC*_NS_) and TREK-1 (*IMSC*_K_), based on (Xian et al., 2006) with parameters adjusted to reproduce outcomes measured by us *in vitro*. *IMSC*_*NS*_ had a linear current–voltage relationship given by:

$$IMSC_{\text{NS}}=gMSC_{\text{NS}}\frac{\lambda }{10}\left(V-EMSC_{\text{NS}}\right)$$

where *gMSC*_NS_ = 200 pS is the current maximal conductance with 10% stretch, *λ* is the magnitude of the applied stretch (20% in this study) and *EMSC*_NS_ = −10 mV is the current reversal potential. *IMSC*_*K*_ is modelled as an outwardly-rectifying current:

$$IMSC_{K}=\frac{gMSC_{K}\frac{\lambda }{10}}{1+\text{exp}\frac{30-V}{15}}$$

with *gMSC*_*K*_ = 2500 nS. Note that our formulations result in *IMSC*_K_ being significantly larger than *IMSC*_NS_ at positive potentials, with the absolute magnitudes of the two currents being similar at resting potentials, as predicted experimentally by (Xian et al., 2006).

**Fig. 10:**
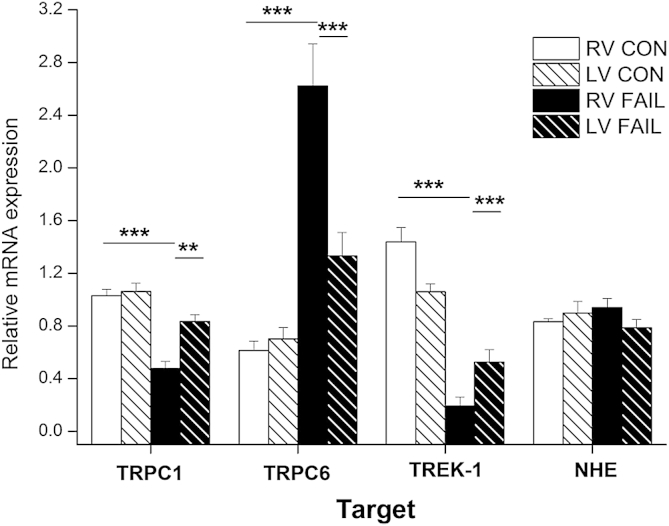
Real-time RT-PCR measurement of mRNA for proteins thought to represent non-selective cationic mechanosensitive ion channels (TRPC1 and TRPC6), a potassium selective mechanosensitive ion channel (TREK-1) and a mechanosensitive ion exchanger (NHE). Expression is given for myocardium from RV and LV of CON and FAIL hearts. Data expressed relative to a calibrator sample and normalised to the housekeeper gene 18S. In the FAIL RV there was a statistically significant decrease in TRPC 1 and TREK-1 but an increase in TRPC6 compared to CON RV (*N* = 10 CON and 12 FAIL hearts). **P < 0.01; ***P < 0.001.

To simulate MSCs in FAIL, we reduced g*MSC*_NS_ by 11% and g*MSC*_K_ by 87% based on mRNA changes (Fig. 10) and TRPC 1:TRPC6 abundance which was scaled at 8:1 based on the cycle threshold in real time RT-PCR experiments being 3 higher in TRPC6 (3^2^ = 8 fold reduced initial copy number). Action potentials were recorded after a train of 10 stimuli were applied at 5 Hz with a 5 nA stimulus current of 1 ms duration. For simulations with stretch, we applied 20% stretch immediately prior to the final stimulus. The level of stretch was an estimation of the change in length reported to occur from slack length (SL 1.8–1.9 μm) to the peak of the length-tension relationship (SL 2.2–2.3 μm).

## 3 Results and discussion

### 3.1 Alternans: *in vivo* to whole heart

*In vivo* recordings of ECG by telemetry from conscious, unrestrained FAIL animals revealed T-wave alternans in a sub-set of animals (Fig. 1A). T-wave alternans are thought to reflect underlying alternating patterns of ventricular repolarisation. Optical mapping of electrical activity from isolated whole hearts revealed discordant APD alternans, (alternans with regions out of phase, Fig. 1B). APD alternans are more likely to arise when there is increased dispersion of repolarisation (Fig. 1C, D) and/or steeper APD restitution (Fig.1E): both conditions were found in MCT-treated hearts.

It was observed that while the APD in the Right Ventricular Outflow Tract (RVOT) region was typically shorter than the APD measured midway between RV apex and base (mid-RV) in CON rats (in 11/11 hearts) it was significantly longer in MCT hearts (in 8/9 hearts) (Fig. 1C, D). This is interesting because it suggests re-modelling is greater in the RVOT, a region where many arrhythmic syndromes and the majority of idiopathic ventricular arrhythmias are found. A longer APD coupled with the late activation of the RVOT is also likely to increase the dispersion of repolarisation.

Structural remodelling can affect electrical conduction and be pro-arrhythmic in its own right (Engelman et al., 2010). DT-MRI was used to measure the rotation of myocardial fibre angles across the RV wall (Fig. 2A). In 3D DT-MRI reconstructions, there were no changes to LV structure with FAIL, but mean RV wall thickness was increased from 1.6 to 2.2 mm and the rate of transmural fibre rotation decreased from 148 to 86°/mm. The absolute change in angle was not different between CON and FAIL hearts, but the thicker RV wall meant that the rate of change of angle was less in FAIL hearts, decreasing from 73 ± 7.8°/mm to 37 ± 3.6°/mm. In addition the correlation between fibre angle and position in the RV wall was much weaker in FAIL hearts (*R*^2^ = 0.72 ± 0.05 for CON and 0.46 ± 0.07 for FAIL) suggesting a greater heterogeneity of fibre orientation (Fig. 2B, and see (Benoist et al., 2012).

Ventricular dimensions and fibre angle rotation were combined with a simulation of electrical activity based on relative mRNA gene expression, the APD_90_ increased in FAIL from 36 to 51 ms in LV cells and from 31 to 78 ms in RV cells. Following programmed stimulation in the RV wall, we found an increased propensity for transition to ventricular fibrillation in FAIL, with the structural and functional changes playing a synergistic role in this increase (Fig. 2C, D) suggesting that the transition in FAIL is dependent on both structural and functional remodelling.

### 3.2 Alternans: single cell to gene expression

There is good evidence that electrical alternans are associated with Ca^2+^-handling anomalies. The varying Ca^2+^ transient amplitude is thought to generate varying inward Na–Ca exchange current which in turn modulates APD. When stimulation frequency was elevated above 5 Hz, Ca^2+^ transient alternans were seen in myocytes from FAIL but not CON hearts (Fig. 3A). Ca^2+^ transient alternans have been linked to decreased function of Ca^2+^ cycling by the sarcoplasmic reticulum Ca^2+^ uptake pump (SERCA). In FAIL myocytes there was decreased SERCA activity, assessed by measuring *K*_SERCA_, the rate constant of Ca^2+^ transient decay in the presence and absence of SR Ca^2+^ accumulation (Fig. 3B). It should be noted however that estimates of K_SERCA_ based in part on the decay of electrically stimulated Ca^2+^ transients will be influenced by changes in action potential repolarisation time and it is well established that the FAIL action potential is longer than the CON action potential. Consistent with decreased *K*_SERCA_ was decreased SERCA protein, measured by Western Blot (Fig. 3C) and mRNA levels of the gene expressing SERCA (Fig. 3E) measured by real-time RT-PCR. Interestingly in the HYP group, levels of SERCA protein were negatively correlated with RV hypertrophy (Fig. 3D).

SERCA activity is decreased by Phospholamban (PLB): this inhibition is relieved by phosphorylation of PLB. We observed a decrease in both the mRNA (Fig. 4A) and protein (Fig. 4B) for PLB in FAIL hearts. Levels of PLB phosphorylation at the Serine 16 (PKA-dependent site) and Threonine-17 (Ca-Calmodulin dependent site) were reduced (Fig. 4C, D). The decrease in phosphorylated Serine 16 was not different to decrease in total PLB (Fig. 4E). The decrease in phosphorylated Threonine 17 was less than the decrease in total PLB (Fig. 4F). Some models of heart failure show decreased levels of SERCA in the presence of maintained PLB and explain decreased SERCA function in terms of altered SERCA:PLB. We did not find such a relationship in the MCT model and conclude the decreased levels of SERCA are the main reason for its decreased function.

The above section of the article is focussed on the demonstration of a systems approach to study alternans and we have not given a full account of arrhythmogenic mechanisms in the MCT model. One important property not dealt with is conduction velocity. This is slowed in FAIL hearts, relative to CON hearts, as stimulation increases (Benoist et al., 2012). Thus, conduction velocity restitution is steeper in FAIL hearts and this could contribute to re-entry type arrhythmias.

### 3.3 Mechanical stimulation in MCT hearts

Table 1 gives whole animal and organ characteristics of the CON and FAIL animals used in the investigation of mechanical modulation. In agreements with previous studies there was an increase in heart weight:body weight and lung weight:body weight in FAIL animals compared to CON. The increase in HW:BW was principally due to RV hypertrophy, indexed as a significant increase in RV:LV weight.

**Table 1:** Whole animal and organ weights for CON and FAIL animals used in the mechanical stimulation studies. RV (right ventricle) LV (left ventricle).

	CON (*N* = 34)	FAIL (*N* = 29)
Body weight (g)	332 ± 6	279 ± 5***
Heart weight (g)	1.53 ± 0.05	1.68 ± 0.05*
RV weight (g)	0.23 ± 0.01	0.37 ± 0.02***
LV weight (g)	0.51 ± 0.01	0.46 ± 0.01**
Heart weight:body weight (mg/g)	4.52 ± 0.10	6.00 ± 0.15***
RV weight:LV weight (mg/mg)	0.46 ± 0.02	0.81 ± 0.04***
Lung weight:body weight (mg/g)	5.31 ± 0.21	9.37 ± 0.34***
Liver weight:body weight (mg/g)	40.83 ± 0.71	43.41 ± 0.99 (*P* = 0.06)

*P < 0.05; **P < 0.01; ***P < 0.001.

Echocardiography from anaesthetised animals was performed in B-mode to calculate the radius of curvature of the RV (Fig. 5A), M-mode to calculate RV wall thickness (Fig. 5B) and Doppler to calculate PAAT (Fig. 5C). Mean data (Fig. 5D) shows greater wall stress in FAIL hearts and thus evidence of chronic increased mechanical stimuli. We previously estimated a 60% increase in wall stress in FAIL hearts (Stones, 2013) based on values from different hearts measured after different experimental procedures. The values presented here were calculated for each heart individually with parameters collected simultaneously. The increase in wall stress is about twice the previous estimate.

### 3.4 Length-dependent changes in tension in single myocytes

To measure the response to acute increase in strain, single RV myocytes were attached to flexible carbon fibres and stretched. Changes in resting and active force were normalised to cell cross- sectional area (XSA). Consistent with RV hypertrophy, the XSA of FAIL myocytes was significantly increased (Fig. 6A). There was a trend for resting SL to be shorter in FAIL myocytes but this was not statistically significant (Fig. 6B). Active force normalised to XSA was not altered (Fig. 6C). Myocytes were stretched and resting SL increased by approximately 0.11 μm (Fig. 6). The increase in tension normalised to SL increase was calculated for resting tension (Fig. 6E) and active tension (Fig. 6F) to give an index of length dependent changes. There were no significant differences between CON and FAIL myocytes. These experiments indicated little effect on the length-tension relationship of FAIL myocytes. However only SLs at the lower end of the length-tension curve were tested due to technical difficulties in maintaining cell attachment of intact myocytes to carbon fibres at longer SL.

To investigate length-tension relationships over a wider range of SL, permeabilised myocytes were glued to force transducers and force-pCa relationships measured at SL of 2.0 and 2.3 μm (Fig. 7). The relationships were fitted with a Hill equation. Mean data for RV myocytes are shown in (Fig. 7A). There was no significant difference in the maximally activated force of CON and FAIL myocytes nor the slope of the relationships from either the LV or RV. In both CON and FAIL myocytes an increase in SL caused a left-shift in the curve, indicative of a length-dependent increase in myofilament sensitivity to Ca^2+^. The pCa at half maximal activation (pCa_50_) is an index of myofilament Ca^2+^ sensitivity and the change in this value (ΔpCa_50_) upon increased SL, an index of the length-dependant change. The ΔpCa_50_ was significantly greater in CON RV than LV but there was no difference between CON and FAIL (Fig. 7B). There are reports of myofilament Ca^2+^ sensitivity being increased, decreased and unchanged in heart failure. The effect of heart failure on the Frank-Starling mechanism is also subject of debate with studies finding the mechanism unaltered or depressed (von Lewinski et al., 2009). We have found no evidence of a change in myofilament Ca^2+^ sensitivity or in its response to length changes in FAIL myocytes in this model.

### 3.5 Stretch activated arrhythmias in CON and FAIL hearts

Langendorff-perfused isolated whole hearts were stimulated at a frequency of 5 Hz and MAPD was monitored in response to an acute stretch to the volume that gave maximum active force development in each heart. The FAIL hearts were larger than CON hearts and this volume was greater (CON 78.3 ± 7.0 μl, *N* = 6; FAIL 101.3 ± 8.3 μl, *N* = 8, *P* < 0.05). Stretch-induced disruption of rhythm was seen in both groups immediately following stretch (Fig. 8A). Rhythmicity was assessed as the standard deviation (SD) of the beat to beat interval prior and immediately following the stretch. The stretch-induced disruption to rhythm (increase in SD of the beat to beat interval) was smaller in the FAIL hearts (Fig. 8B).

As previously reported, MAPD was longer in the RV of FAIL animals (Fig. 9A). When stable rhythm was re-established following a stretch, MAPD at 25% repolarisation was reduced in CON hearts but not FAIL hearts. There was no effect on MAPD at 50% or 90% repolarisation in either group (Fig. 9B).

These observations were somewhat unexpected given that the MCT model has a pro-arrhythmic substrate and acute stretch is an acknowledged arrhythmic stimulus. It therefore seemed likely these 2 factors would operate synergistically when FAIL hearts were stretched. An additional factor may be that our stretch-stimulus was not sufficient to generate sustained tachycardias or fibrillation and in this situation the index of rhythmicity is linked to refractoriness. It is known that the refractory period of FAIL hearts is longer than CON hearts (Benoist et al., 2011), probably because of the longer APD. The longer the refractory period the lower the maximal rate of excitation and the lower the possible range of beat to beat intervals.

The observation that in CON, APD_25_ was shortened by stretch could be explained in terms of the activation of MSC_NS_ and MSC_K_. At the elevated membrane potentials of the early AP both these channels, if active, would be predicted to generate outward repolarising current that would speed repolarisation. However, during late repolarisation the membrane potential would fall below the predicted equilibrium potential of MSC_NS_, generating an inward current that would oppose MSC_K_ which would in turn decay as the membrane potential approached the equilibrium potential for K^+^. The net effect could be no change in MAPD_90_, as observed.

Data from whole hearts suggested that the electrical response to acute stretch was not enhanced and possibly decreased in FAIL hearts. If electrical changes were dependent upon MSC, this might indicate reduced levels of MSCs in MCT hearts. To test this possibility we measured the expression of mRNA for the genes encoding channels thought to be responsible for MSC_NS_ (TRPC 1 and 6) and MSC_K_ (TREK-1) using real time RT-PCR. In addition we measured expression of the Na–H exchanger (NHE). Though not electrogenic, NHE has been implicated in the generation of stretch-activated current via alterations in [Na^+^]i. In the RV of FAIL hearts there was a decrease in the mRNA levels for TRPC-1 and TREK-1 but an increase in TRPC6 compared to both RV CON and LV FAIL. NHE mRNA levels were unchanged (Fig. 10).

**Fig. 11:**
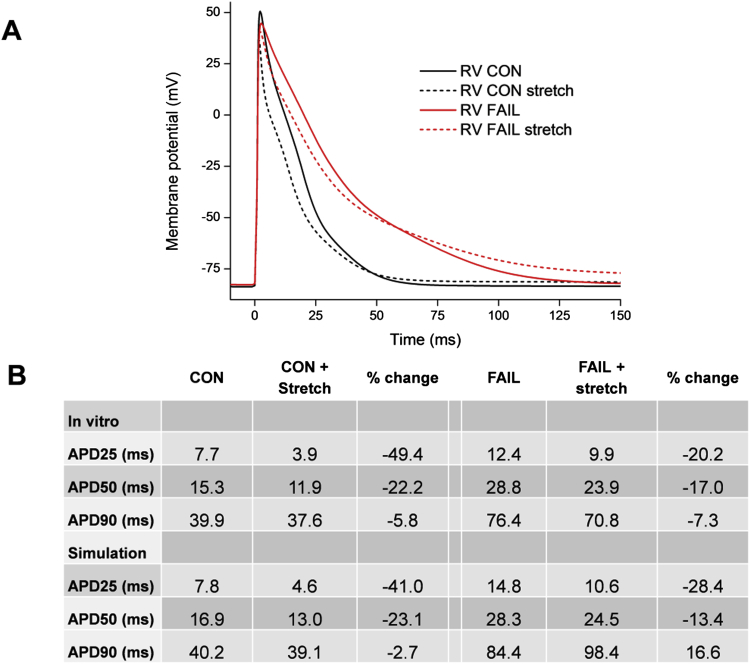
A Computer simulation of the effect of activation of mechanosensitive channels (MSCs) on the action potentials of CON and FAIL hearts. B. Data for mean APD at 25, 50 and 90% repolarisation from *in vitro* experiments and from computer simulation. CON and FAIL APD and MSCs were simulated as described in the Methods and included MSC_NS_ based on TRPC1/6 and MSC_K_ based on TREK-1. A simulated 20% stretch shortened the CON action potential with a greater effect in early repolarisation (when both MSC_NS_ and MSC_K_ pass repolarising current) than later. The repolarising effect of stretch was less apparent in FAIL where MSC_NS_ was reduced by 11% and MSC_K_ by 87% based on mRNA measurement of genes expressing TRPC1/6 and TREK-1.

Computer simulation of the activation of MSC_NS_ and MSC_K_ were performed (Fig. 11A). Using the parameters described in the Methods, based on published data for MSC_NS_ and MSC_K_ together with our mRNA-based remodelling data for FAIL, it was possible to reproduce stretch effects that closely mimic our *in vitro* observations. Stretch shortened the CON rat APD with a greater effect (early in repolarisation) than in FAIL hearts (Fig. 11B). An interesting observation from the simulation is that in FAIL reduction in MSC_K_ with relatively maintained levels of MSC_NS_ generates a net depolarising effect compared to CON. *In vitro* measurement or computer simulation of either current in isolation would not have identified this interaction.

Thus the decreased responsiveness of FAIL hearts to stretch may be linked to the decreased expression of MSCs and computer simulations suggest this is a feasible possibility. Decreased expression of MSCs in PAH rats may be a response to the chronic pressure and volume overload that occurs, in order to prevent excessive activation of MSCs.

## 4 Conclusion

The technologies we have used are not novel *per se* but in several instances their combination is for example; the use of DT-MRI, whole heart electrophysiology and real time RT-PCR to create a structural and electrical simulation of the PAH rat heart. The use of multiple methodologies at different levels has enabled us to demonstrate mechanisms associated with the generation of alternans from *in vivo* function to protein expression and function and to make links that could not have been made by studying a single level. In practice the experimental process was not linear, the observation of Ca^2+^ transient alternans in single myocytes and steep APD restitution in isolated hearts led to further studies, both reductionist and integrative in nature. In our investigation of mechanical stimulation, using the 3 techniques; MAP recording, mRNA measurement and computer simulation give a better understanding of the phenomena (and provide synergistic evidence for a mechanism) than would have been available from a single technique. Incorporation of *in vivo* wall stress data and MSC expression into our simulation is a future possibility. What is apparent from our studies is that it is highly unlikely to find an individual scientist with the skills and time to perform all the techniques discussed in this study, therefore collaboration is both welcome and necessary, ‘systems biology’ is a team sport.

## Editors’ note

Please see also related communications in this issue by Ravelli et al. (2014) and Rouillard et al. (2014).
